# Sintilimab plus cisplatin and nab‐paclitaxel induction treatment for locally advanced borderline‐resectable oesophageal squamous cell carcinoma: A single‐arm, prospective, phase 2 study (NEOCRTEC2001)

**DOI:** 10.1002/ctm2.70691

**Published:** 2026-05-11

**Authors:** Jia‐Di Wu, De‐Shen Wang, Zhi‐Qiang Wang, Qiao‐Qiao Li, Bin‐Yang Xu, Chao Ren, Cai‐Yan Fang, Yan Huang, Zhi‐Chao Li, Ji‐Yang Chen, Qiong Tan, Yu‐Hong Li, Hong Yang

**Affiliations:** ^1^ State Key Laboratory of Oncology in South China Guangdong Provincial Clinical Research Center for Cancer Sun Yat‐sen University Cancer Center Guangzhou P. R. China; ^2^ The First Clinical Medicine College Gansu University of Chinese Medicine Lanzhou P. R. China

**Keywords:** borderline‐resectable oesophageal squamous cell carcinoma, conversion surgery, immunochemotherapy

## Abstract

**Background:**

The standard treatment for locally advanced borderline‐resectable esophageal squamous cell carcinoma (BR‐ESCC) is still debated owing to insufficient evidence from clinical trials. An increasing number of clinical studies focus on investigating the use of immunotherapy in the treatment of oesophageal cancer. This phase II trial (NEOCRTEC‐2001) aimed to assess the safety and efficacy of sintilimab in combination with cisplatin and nab‐paclitaxel induction immunochemotherapy followed by surgery for BR‐ESCC.

**Methods:**

The NEOCRTEC2001 trial was a single‐centre, open‐label, nonrandomized, phase II study. Patients diagnosed with BR‐ESCC were enrolled in the study and initially received 2–4 courses of induction immunochemotherapy at first. The subsequent treatment, surgery or definitive chemoradiotherapy, was determined based on reassessment by MDT. The primary endpoint of the study was the R0 resection rate.

**Results:**

From September 2020 to June 2024, a total of 50 eligible patients diagnosed with BR‐ESCC were enrolled. All eligible patients underwent induction immunochemotherapy as the initial treatment. After induction immunochemotherapy, 35 of 50 patients (70.0%) were considered resectable, and 29 patients (58.0%) underwent surgery. R0 resection was achieved in 28 patients (56.0%, 95% CI, 41.4–69.1%), and 9 patients (18.0%) achieved pathological complete response. The median follow‐up time of all patients was 29.43 months. Patients in the R0 resection group demonstrated significantly superior overall survival (OS) and progression‐free survival (PFS) compared to those in the non‐R0 group (OS: not reached vs. 19.84 months; HR  .25; 95%CI  .08–.79, *p* = .001; PFS: not reached vs. 19.82 months; HR  .30; 95%CI  .10–.90, *p* = .006).

**Conclusions:**

The regimen under investigation did not exhibit the anticipated statistical benefit in enhancing surgical conversion rates for BR‐ESCC. Nevertheless, the treatment strategy of induction immunochemotherapy followed by surgery resulted in significant tumour downstaging and a significant pathological complete response rate. Patients who achieved R0 resection exhibited improved survival outcomes.

**Trial registration:**

The study was registered at ClinicalTrials.gov (NCT04548440)

**Key points:**

- To the best of our knowledge, this is the first trial to evaluate the sintilimab combined with chemotherapy as an induction treatment for patients with BR‐ESCC. The R0 resection rate in this study was 56.0%, and the pCR rate was 18.0%. Patients who achieved R0 resection exhibited improved survival outcomes.

## Graphical abstract

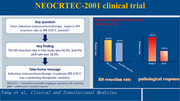

## 1 BACKGROUND

Esophageal squamous cell carcinoma (ESCC) represents the predominant histological subtype of oesophageal cancer in the Asian population, accounting for more than 90% of patients.^1^, ^2^ For locally advanced resectable ESCC, the standard treatment involves neoadjuvant therapy in conjunction with radical surgery. Conversely, definitive chemoradiotherapy (dCRT) is considered the standard treatment for locally advanced unresectable ESCC.^3^, ^4^, ^5^ Owing to the lack of a serosal layer, ESCC easily invades adjacent structures such as the trachea, bronchus, and aorta. Patients whose primary tumour or lymphadenopathy is suspected of organ involvement, but lacks a definitive diagnosis of cT4b are considered as borderline‐resectable esophageal squamous cell carcinoma (BR‐ESCC).^6^, ^7^ Recent studies have investigated various treatment strategies for this subset of patients. However, the optimal treatment approach remains contentious.^8^, ^9^, ^10^, ^11^

In a previous NEOCRTEC1601 trial,^6^, ^7^, ^12^ the aim was to assess the efficacy and safety of paclitaxel, cisplatin and 5‐fluorouracil (TPF) as an induction chemotherapy for BR‐ESCC. Between July 2014 and February 2019, a total of 47 patients diagnosed with BR‐ESCC were enrolled in the study. The results of the trial indicated that 53.2% of patients achieved R0 resection, and 8.5% of patients were confirmed to have a pathologic complete response (pCR). Long‐term follow‐up evaluation confirmed that the overall survival (OS) and progression‐free survival (PFS) were significantly better in patients who underwent R0 resection than in those who did not. The 5‐year OS rate was 50.0% in patients who underwent R0 resection and 19.0% in those who did not (HR  .36, 95%CI:  .17–.79, *p* = .0041). In addition, R0 resection might be an independent prognostic factor for OS.

Immunochemotherapy has emerged as the standard first‐line treatment for advanced oesophageal cancer. Preliminary findings from phase II/III trials indicate that incorporating immunotherapy into neoadjuvant treatment yields a promising pCR rate in patients with locally advanced resectable ESCC.^13^, ^14^, ^15^ The ORIENT‐15 trial^16^ demonstrated that the combination of sintilimab and chemotherapy significantly improved the overall survival in patients with advanced or metastatic ESCC compared to chemotherapy alone (median time 16.7 vs. 12.5 months, HR  .63, 95% CI  .51–.78, *p* < .001). In addition, sintilimab appears to confer benefits to all patients, irrespective of PD‐L1 expression levels, without increasing adverse events. Consequently, it is plausible to hypothesize that immunochemotherapy might improve the therapeutic efficacy of BR‐ESCC.

In this study, we have designed a prospective phase II trial to evaluate the antitumor activity and safety of induction immunochemotherapy (regimen: sintilimab + nab‐paclitaxel + cisplatin) followed by surgery or definitive chemoradiotherapy in patients with BR‐ESCC.

## 2 METHOD

The study received approval from the Ethics Committee of Sun Yat‐sen University Cancer Center. All patients included in the study provided written informed consent before enrolment. Furthermore, the study was registered at ClinicalTrials.gov (NCT04548440).

### 2.1 Patients

Patients with histologically confirmed BR‐ESCC were recruited for this study. BR‐ESCC was characterized by primary tumours or bulky lymphadenopathy suspected of invading adjacent structures, including the aorta, arch vessels, airway, and vertebral body. The specific diagnostic criteria were as follows: (1) Computed tomography (CT) revealed an indistinct fat plane between the tumour and the aorta, with an angle exceeding 90° between the tumour and the aorta across three consecutive slices (2 mm/slice). (2) Endoscopic ultrasound (EUS) revealed that the tumour had penetrated the outer membrane layer, with an indistinct boundary from the aorta. (3) Endobronchial ultrasound (EBUS) revealed that the tumour had an indistinct boundary with the trachea or bronchus, but did not invade the mucosal or submucosal layers of the trachea or bronchus. Other inclusion and exclusion criteria are presented in the Supporting Information.

### 2.2 Study design and end points

The NEOCRTEC2001 trial was a single‐centre, open‐label, nonrandomized, phase II study. The primary endpoint of the study was the R0 resection rate. The secondary endpoints of the study included the pCR rate, OS, PFS, adverse events (AEs), postoperative complications and pathological response.

### 2.3 Treatment procedure

The protocol therapy started with 2–4 cycles of induction treatments every 3 weeks. Following two cycles of chemotherapy combined with immunotherapy, patients undergo a restaging evaluation. Individuals who satisfy the criteria for resectability advance to radical resection. Conversely, patients exhibiting no response in their lesions are administered definitive chemoradiotherapy. For those whose lesions have regressed but have not yet fully met the resectability criteria, additional cycles of preoperative therapy are provided. Subsequently, a radiological evaluation and multidisciplinary assessment will be conducted. If radical resection was possible, surgery was performed 4–6 weeks after the last chemotherapy session. In the case of an R0 resection, the decision to administer adjuvant therapy was made by the investigator based on the patient's condition. In the case of R1 or R2 resection, concurrent chemoradiotherapy was recommended. If the multidisciplinary assessment concluded that radical resection was not feasible, definitive chemoradiotherapy (dCRT) was implemented.

### 2.4 Induction immunochemotherapy

The induction treatment included 2–4 cycles of sintilimab plus nab‐paclitaxel and cisplatin (Sintilimab: 200 mg IV on Day 1; Albumin‐bound paclitaxel: 125 mg/m^2^ IV on Day 1 and Day 8; Cisplatin: 75 mg/m^2^ IV on Day 1; Standard hydration regimen on Day 0–3) every 3 weeks. The toxic effects of chemotherapy were assessed according to the National Cancer Institute's Common Terminology Criteria for Adverse Events (CTCAE), version 4.0.

### 2.5 Surgery

For patients deemed resectable based on clinical restaging, surgery was scheduled 4–6 weeks after the completion of induction immunochemotherapy. A thoracoscopy combined with a laparoscopy McKeown esophagectomy, along with a two/three‐field lymphadenectomy, was performed. The McKeown procedure was typically recommended due to its advantages in achieving total mediastinal lymph node dissection, especially for the dissection of left and right recurrent laryngeal nerve lymph nodes. After the resection of the primary tumour, the alimentary tract was reconstructed using an end‐to‐side technique between the oesophagus and the gastric conduit or colon. Enteral nutrition was provided via a feeding tube inserted into the jejunum through the jejunostomy, or via a nasal feeding tube. Routinely, the nasogastric tube was removed 1 day after confirming the absence of anastomotic haemorrhage. Enteral nutrition started on the first postoperative day, and modified oral feeding was allowed after confirming the absence of anastomotic leakage by an oral contrast study or esophagogastroduodenoscopy. Postoperative complications were graded according to the Clavien–Dindo classification.

### 2.6 Postoperative pathology

Residual tumour evaluations were categorized into three classifications: R0 resection (no microscopic or macroscopic residual tumour), R1 resection (only microscopic residual tumour), and R2 resection (macroscopic residual tumour). A condition without grossly and microscopically viable tumour in the entire surgical specimen, including the primary tumour site and any resected lymph nodes, was defined as a pCR.

The tumour regression grade (TRG) was quantified using the scoring system for tumour response of the NCCN guidelines for esophageal cancer^3^: TRG 0 (Complete response: No viable cancer cell, including lymph nodes), TRG 1 (Near complete response: Single cells or rare small groups of cancer cells), TRG 2 (Partial response: Residual cancer with evident tumour regression but more than single cells or rare small groups of cancer cells), and TRG 3 (Poor or no response: Extensive residual cancer with no evident tumour regression).

### 2.7 Definitive concurrent chemoradiotherapy

If the lesion remained unresectable after induction immunochemotherapy, dCRT was administered as the primary treatment modality. The dCRT regimen consisted of administering a total radiation dose of 60–64 Gy over 30–32 fraction, in conjunction with weekly doses of paclitaxel at 50 mg/m^2^ and cisplatin at 25 mg/m^2^.

### 2.8 Sample size

The sample size estimation for the R0 resection rate was conducted based on the target population. Drawing from our prior studies^6^, ^7^ and clinical expertise, the current R0 resection rate for the target population following triplet chemotherapy is approximately 50%. It is anticipated that the R0 resection rate will increase to 70% in the target population when treated with a combination of PD‐1 monoclonal antibody and chemotherapy. Using a statistical power of 0.8 and an exact one‐sided probability test with a significance level (α) of 0.05, the sample size was determined using Simon's two‐stage method, resulting in a requirement for 43 cases. Accounting for an anticipated 15% dropout rate, a total of 50 participants were necessary for enrolment.

### 2.9 Outcome

The primary endpoint of the study was the rate of R0 resection. The secondary endpoints encompassed the pCR rate, OS, PFS, adverse events (AEs), postoperative complications and pathological response. OS was defined as the interval from the date of registration to the date of death from any cause. PFS was defined as the interval from the date of registration to the date of either disease progression or death from any cause.

### 2.10 Statistical analysis

The Chi‐square test or Fisher's exact test, along with Student's *t*‐test, were employed to assess the difference in clinical features between the groups. OS and PFS were evaluated using the Kaplan–Meier method, with comparisons of survival curves conducted via the log‐rank test. Two‐sided *p*‐values less than 0.05 were considered statistically significant. Statistical analyses were conducted using IBM SPSS version 29.0 (IBM Corp.) and R version 4.3.2 (The R Project for Statistical Computing).

## 3 RESULTS

### 3.1 Patient characteristics

From September 2020 to June 2024, a total of 50 eligible patients with BR‐ESCC were treated at the Sun Yat‐sen University Cancer Center. Baseline clinical characteristics are shown in Table 1. The median age of all patients was 61 years (range 46–76 years), and males accounted for 88.0%. The primary tumours in 40 patients (80.0%) located in the middle third of thoracic oesophagus. There are 19 patients (38.0%) underwent two cycles of induction treatments, 1 patient received only one cycle due to severe AEs, and 30 patients (60.0%) underwent more than two cycles of induction treatment. The R0 resection rate and pCR rate in patients who received more than 2 courses of induction therapy did not show a significant improvement. (R0 resection rate: 50.0% (≤2 cycles) vs. 60.0% (> 2 cycles), *p* = .567; pCR rate: 40% (≤2 cycles) versus 26.5% (> 2 cycles), *p* = 0.675). There was no statistically significant difference between the R0 group and non‐R0 group in terms of sex, BMI, smoking history, tumour location, clinical TNM stage or number of cycles of induction treatment.

**TABLE 1:** Baseline characteristics of the intention‐to‐treat population.

Characteristic	All (*n* = 50, %)	R0 group (*n* = 28, %)	Non‐R0 group (*n* = 22, %)	*p*‐value^*^
Age				.003
Median	61	59	65.5	
range	46–76	46–74	55–76	
Sex				.683
Male	44 (88.0)	24 (85.7)	20 (90.9)	
female	6 (12.0)	4 (14.3)	2 (9.1)	
BMI				.683
< 24	44 (88.0)	24 (85.7)	20 (90.9)	
≥24	6 (12.0)	4 (14.3)	2 (9.1)	
Smoking history				.374
Yes	26 (52.0)	13 (46.4)	13 (59.1)	
No	24 (48.0)	15 (53.6)	9 (40.9)	
Tumour location			
Upper	5 (10.0)	3 (10.7)	2 (9.1)	
middle	40 (80.0)	23 (82.1)	17 (77.3)	
lower	5 (10.0)	2 (7.1)	3 (13.6)	
Clinical T stage				na
T4‐br^a^	50 (100.0)	28 (100.0)	22 (100.0)	
Clinical N stage				.092
0	3 (6.0)	1 (3.6)	2 (9.1)	
1	9 (18.0)	7 (25.0)	2 (9.1)	
2	23(46.0)	15 (53.6)	8 (36.4)	
3	15 (30.0)	5 (17.9)	10 (45.5)	
Clinical stage				na
IVA	50 (100.0)	28 (100.0)	22 (100.0)	
Cycle of induction treatment				.628
1	1 (2.0)	0 (0.0)	1 (4.5)	
2	19 (38.0)	10 (35.7)	9 (40.9)	
3	26 (52.0)	16 (57.1)	10 (45.5)	
4	4 (8.0)	2 (7.1)	2 (9.1)	

Abbreviation: BMI, body mass index.

^a^ T4‐br: All patients met our predefined diagnostic criteria for BR‐ESCC.

* Compare R0 group and non‐R0 group.

### 3.2 Primary endpoint and treatment overview

**FIGURE 1:**
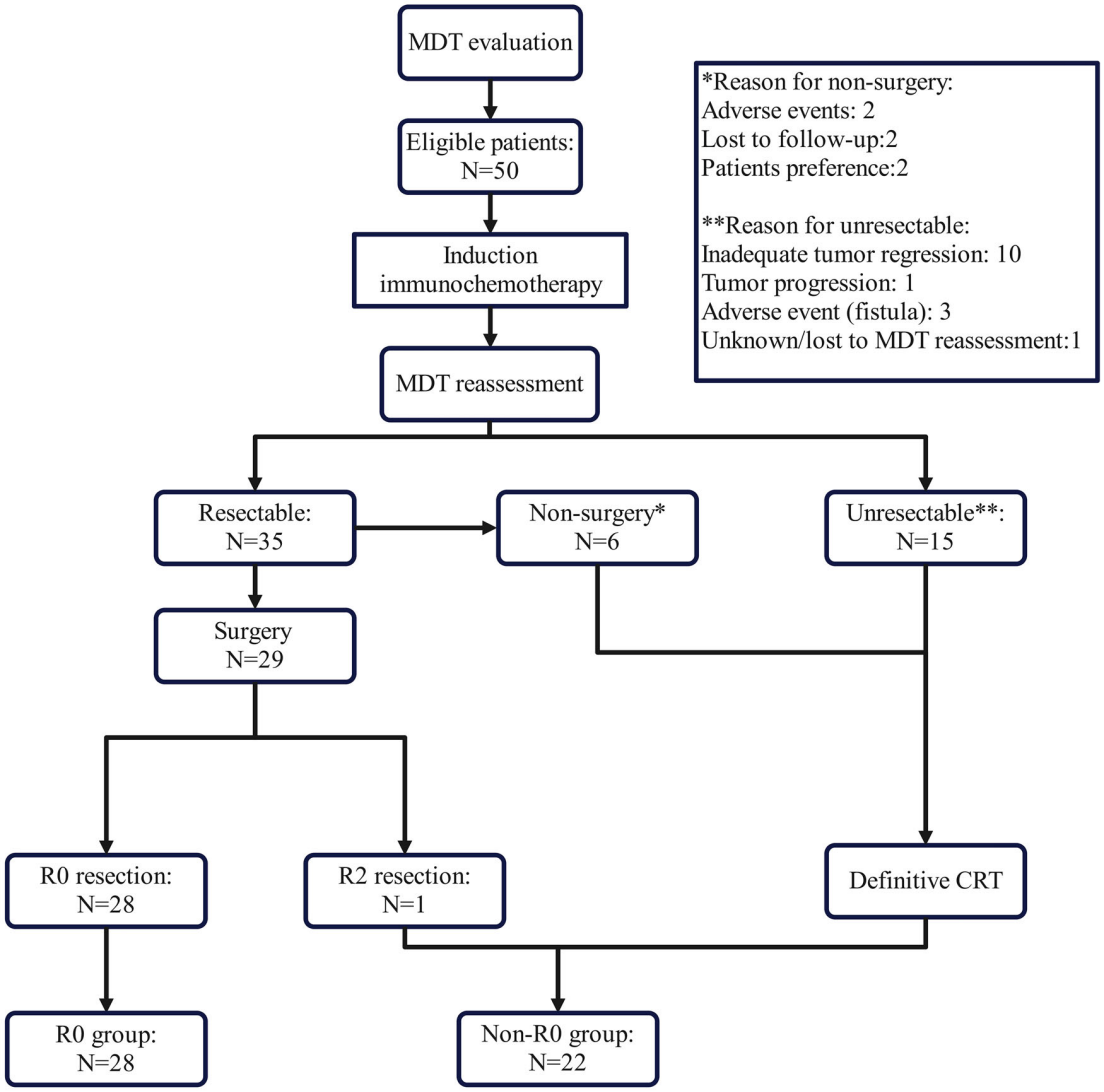
Consort flow diagram.

Figure 1 provides an overview of the treatment administered to all patients. All eligible patients underwent induction immunochemotherapy as the initial treatment. After induction immunochemotherapy, 35 of 50 patients (70.0%) were considered as resectable and 29 patients (58.0%) underwent surgery. R0 resection was achieved in 28 patients (56.0%, 95% CI, 41.4%–69.1%) and 1 patient (2.0%) underwent R2 resection. In addition, 9 patients achieved pathological complete response, and the pCR rate of the ITT patients was 18.0%. The reasons for the 6 patients who were diagnosed as resectable but did not undergo surgery included severe adverse events (*n* = 2: Grade 3 immunotherapy‐related hepatitis; Grade 3 immunotherapy‐related hypothyroid combined with diabetes), patient preference (*n* = 2), and loss to follow‐up (*n* = 2). The reasons for the 15 patients (30.0%) who were considered as unresectable included inadequate tumour regression (*n* = 10), tumour progression (*n* = 1), adverse events (*n* = 3: Grade 3 fistula), and loss to MDT reassessment (*n* = 1). Figure S1 presents the endoscopic and CT images before and after induction therapy of a typical pCR patient.

### 3.3 Adverse events associated with induction immunochemotherapy

Table 2 presents the adverse events observed during induction immunochemotherapy. A total of 46 patients (92.0%) experienced adverse events of varying severity, with haemoglobin decrease (64.0%) and weight loss (46.0%) being the most frequently observed AEs. AEs of Grade 3 or higher were observed in 8 patients (16.0%). The incidence of AEs was similar between patients who underwent more than 2 courses and those who received only two courses (93.3% vs. 90.0%, *p* = 1.00). The most common serious AEs (≥Grade 3) included immunotherapy‐related pneumonia (4.0%), immunotherapy‐related hypothyroid or hypocortisolism (4.0%), and fistula (6.0%).

**TABLE 2:** Adverse Events during immunochemotherapy (*N* = 50, %).

Adverse Event	Any grade	Grade 1–2	Grade 3	Grade 4
Weight loss	23 (46.0)	23 (46.0)	0	0
Leukopenia	13 (26.0)	13 (26.0)	0	0
Neutropenia	13 (26.0)	12 (24.0)	1 (2.0)	0
Haemoglobin decrease	32 (64.0)	32 (64.0)	0	0
Aminotransferase increased	5 (10.0)	5 (10.0)	0	0
Albumin decrease	12 (24.0)	12 (24.0)	0	0
Nausea/vomiting	9 (18.0)	9 (18.0)	0	0
Anorexia	9 (18.0)	9 (18.0)	0	0
Rash	3 (6.0)	3 (6.0)	0	0
Fatigue	4 (8.0)	4 (8.0)	0	0
Alopecia	2 (4.0)	2 (4.0)	0	0
Diarrhoea or constipation	7 (14.0)	7 (14.0)	0	0
Immunotherapy‐related pneumonia	2 (4.0)	0	2 (4.0)	0
Immunotherapy‐related hepatitis	1 (2.0)	0	1 (2.0)	0
Immunotherapy‐related hypothyroid	2 (4.0)	1 (2.0)	1 (2.0)	0
Immunotherapy‐related hypocortisolism	1 (2.0)	0	1 (2.0)	0
Oesophageal fistula	2 (4.0)	0	2 (4.0)	
Oesophagotracheal fistula	1 (2.0)	0	1 (2.0)	0

### 3.4 Surgical details, postoperative complications and pathological results

The surgical details and pathological results of the patients who underwent surgery are presented in Tables 3 and 4. All surgery patients (*N* = 29) underwent totally minimally invasive McKeown surgery, with a median operation time of 275 min and a median blood loss of 50 mm, and no patients required conversion to open thoracotomy or laparotomy. A median of 11 lymph node stations and 47 lymph nodes were dissected per patient. Three patients underwent cervical lymph node dissection. Gastric conduit reconstruction with cervical anastomosis was performed in all cases. The median hospital stay was 14 days. A total of 10 patients (34.4%) were diagnosed with any grade of postoperative complication. In addition, postoperative complications of Grade 3 or higher were observed in 6 patients (20.69%). Among them, 3 cases (30.0%, 3/10) occurred in patients who received 2 cycles of induction therapy, while other 3 cases (15.8%, 3/19) were observed in those with more than 2 cycles, with no statistically significant difference between the two groups (*p* = .663) (Table S1). One patient (3.4%) died within 90 days postoperatively (post discharge, treated at another centre).

**TABLE 3:** Details of surgery.

Data	*N* = 29
median operation time: min (range)	275, 180–489
Median blood loss: mL (range)	50, 30–400
Median No. of LND	47
Median No. of LND stations	11
Cervical LND	3, 6.0%
Median hospital stay: days	14
Surgery options	
McKeown (minimally invasive)	29, 100%
other	0, 0
Anastomotic location	
Neck	29, 100%
Other	0, 0
Substitution	
Stomach	29, 100%
Other	0, 0
Adjuvant immunotherapy	
Yes	11, 37.9%
No	18, 62.1%

**TABLE 4:** Postoperative pathology (*N* = 29).

Evaluations of R	
R0 resection	28, 96.6%
R1/2 resection	1, 3.4%
Stage	
I	20, 69.0%
pCR	9, 31.0%
Non‐pCR	11, 37.9%
II	2, 6.9%
III	7, 24.1%
TRG	
0	12, 41.4%
1	7, 24.1%
2	5, 17.2%
3	5, 17.2%

Among the surgery cohort (*N* = 29), 28 patients (96.6%) achieved R0 resection, whereas one patient was classified as R2 resection due to intraoperative identification of tumour invasion into the aortic arch, which precluded complete resection. Postoperative pathological staging indicated that 20 patients (69.0%) had stage I disease, with 9 of these patients (31.0%) achieving a pCR.

### 3.5 Survival

**FIGURE 2:**
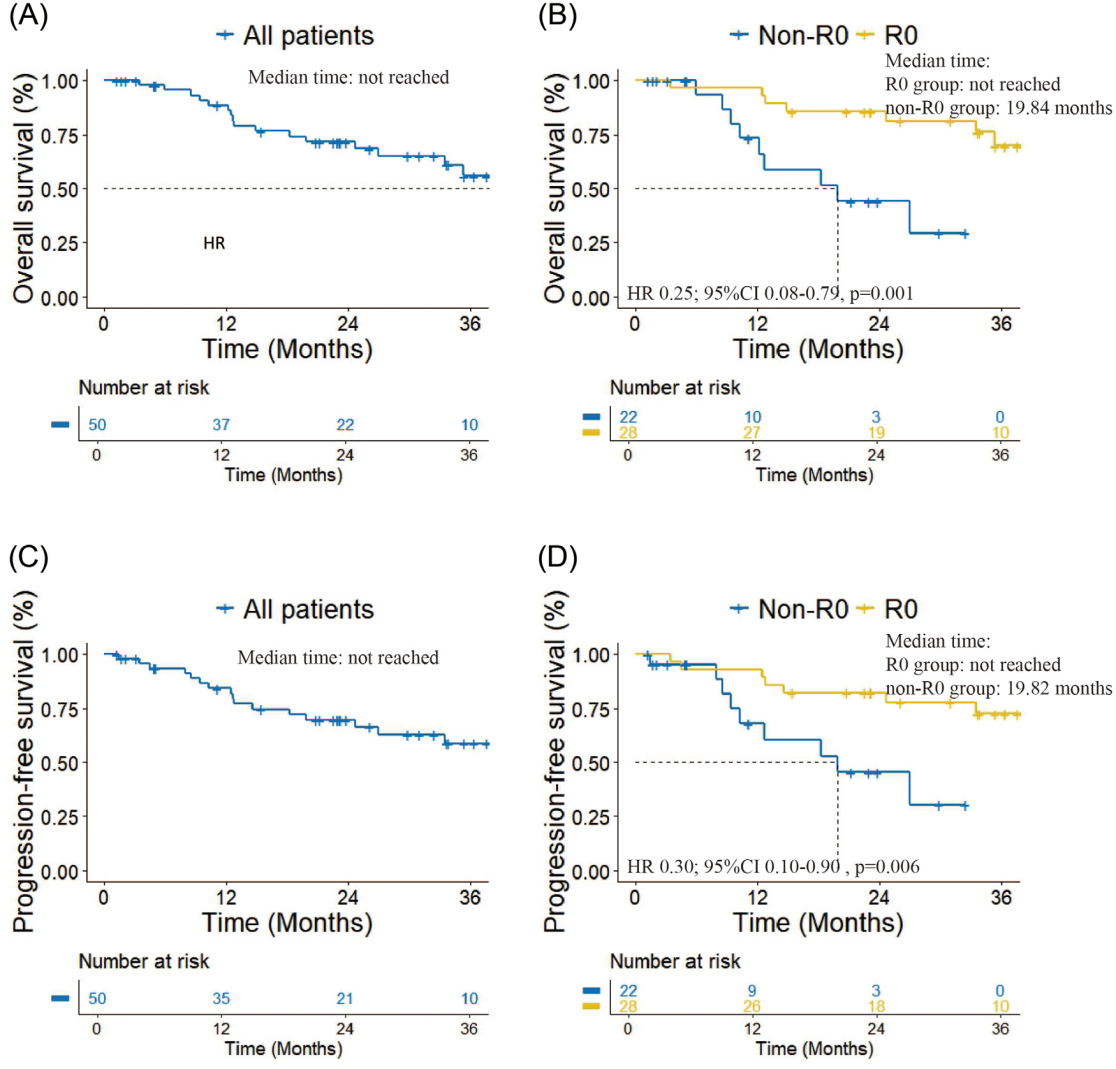
(A) Overall survival of all patients. (B) Overall survival of R0 and non‐R0 group. (C) Progression‐free survival of all patients. (D) Progression‐free survival of R0 and non‐R0 group.

The median follow‐up time of all patients was 29.43 months. Throughout the follow‐up period, a total of 17 patients (34.0%) succumbed, with a distribution of 8 deaths occurring in the R0 group (28.6%) and 9 deaths in the non‐R0 group (40.9%). Within the R0 group, 7 patients died due to tumour progression, and 1 patient died from other causes. In the non‐R0 group, 8 patients died as a result of tumour, and 1 patient died from other diseases. The median OS for all patients was not reached. The OS for the R0 group was significantly longer than non‐R0 group (not reached vs. 19.84 months; HR  .25; 95%CI  .08–.79, *p* = .001). The median PFS for all patients was not reached. The PFS for the R0 group was significantly longer than non‐R0 group (not reached vs. 19.82 months; HR  .30; 95%CI  .10–.90, *p* = .006) (Figure 2).

## 4 DISCUSSION

To the best of our knowledge, this is the first trial to evaluate the sintilimab combined with chemotherapy as an induction treatment for patients with BR‐ESCC. The primary endpoint, the R0 resection rate in this study was 56.0%, which did not meet the prespecified target of 70%. Notably, the study demonstrated a remarkable pCR rate (31% in surgery patients, 18% in all patients) following induction immunochemotherapy. It is noteworthy that increasing the number of induction therapy cycles did not improve the surgery conversion rate. Moreover, 5 patients were unable to undergo surgery due to severe adverse events, highlighting the critical need for toxicity management in induction therapy regimens in subsequent studies. Additionally, this study reinforces the finding that achieving R0 resection can improve OS and PFS in patients with BR‐ESCC.

According to our previous study (the NEOCRTEC1601 trial),^6^, ^7^, ^12^ the OS and PFS were significantly better in patients who underwent R0 resection than in those who did not. The 5‐year OS rate was 50.0% in patients who underwent R0 resection and 19.0% in patients who did not. The 5‐year OS rate for patients with inoperable disease who underwent dCRT was 35.8%.^17^ A noteworthy concern is that whether this induction regimen delays the optimal time window for standard dCRT in patients with a poor response to induction therapy. Firstly, previous NEOCRTEC1601 trial indicated that the integration of induction therapy with radical surgery resulted in a 5‐year survival rate of 50%. Consequently, it is crucial to offer patients with BR‐ESCC treatment strategies that provide enhanced long‐term survival outcomes. Furthermore, in standard clinical practice, the use of induction chemotherapy to reduce tumour burden prior to definitive chemoradiotherapy has become a widely adopted therapeutic approach. R0 resection serves as a crucial prognostic factor for BR‐ESCC. Achieving successful conversion to R0 resection surgery represents a pivotal determinant in the therapeutic management of BR‐ESCC. Recent retrospective cohort studies have demonstrated that induction immunochemotherapy yields an R0 resection rate of 50%–70%.^18^, ^19^, ^20^, ^21^ The BRES‐1 study^22^ was a prospective phase 2 study that enrolled 31 patients with BR‐ESCC. Patients in the study received preoperative immunochemotherapy and conversion surgery. The R0 resection rate of the ITT population was 58.1%. Nevertheless, the study enrolled a part of patients with cM1a staging, who were not categorized as BR‐ESCC according to our trial. In a prospective trial (NEXUS‐1 Trial) conducted by Li,^23^ 30 patients with locally advanced unresectable/borderline‐resectable ESCC were enrolled. Patients were treated with chemoradiotherapy and subsequent immunochemotherapy followed by conversion surgery. The R0 resection rate of the NEXUS‐1 study was 66.7%, and the pCR rate was 43.3%. However, the treatment regimen investigated in this study presents significant challenges for widespread clinical implementation due to its complex therapeutic protocol, prolonged treatment duration and considerable incidence of treatment‐related adverse events. Additionally, compared with our study, these 2 prospective studies had relatively smaller sample sizes, and the R0 resection rate was not used as the primary endpoint. Our study enrolled 50 patients who were diagnosed with BR‐ESCC by MDT assessment. The R0 resection rate observed in our study was 56.0%. The results demonstrated that compared to the TPF chemotherapy regimen, immunochemotherapy did not significantly improve the R0 resection rate, which did not fulfil the predefined study primary endpoint.

The induction therapy regimen must be meticulously optimized to reduce the occurrence of treatment‐related AEs, with a particular emphasis on managing toxicities of Grade 3 or higher, as these can significantly impair subsequent therapeutic interventions and clinical outcomes. The incidence of AEs is approximately 80% in patients receiving triplet chemotherapy, among which severe (≥Grade 3) AEs account for approximately 30%.^6^, ^24^ Recent retrospective cohort studies have demonstrated that induction immunochemotherapy yields a AEs rates of 50%–100%, with severe (≥Grade 3) toxicity in 6%–15% of patients.^18^, ^19^, ^20^, ^21^ In the BRES‐1 trial, the incidence of all‐grade AEs was 100%, and 35.5% of Grade 3 or higher. In our study, 92.0% of patients experienced any grade of adverse events. AEs of Grade 3 or higher were observed in 8 patients (16.0%), which was lower than NEOCRTEC1601 (31.9%). Furthermore, 5 patients (10.0%) were unable to undergo radical surgery due to severe AEs, which was a significant reason contributing to the failure to achieve the predetermined R0 resection rate. Three patients developed fistula during treatment. Fistula caused by rapid tumour regression can occasionally be fatal. Consequently, it is imperative to closely monitor patients throughout the treatment process. In the event of an acute infection, imaging and endoscopic examinations should be conducted promptly to ascertain the presence of a fistula. These findings suggest that future studies should place greater emphasis on monitoring the toxicities associated with induction therapy and their impact on subsequent treatment outcomes. This observation further illustrates the dual nature of immunotherapy in oncology. While it has the potential to induce favourable tumour responses, it may also hinder the efficacy of subsequent treatments due to the occurrence of adverse events.

The existing research has provided substantial evidence demonstrating a significant correlation between R0 resection and prognosis in patients with BR‐ESCC. In the NEXUS‐1 study, the R0 resection group exhibited a longer PFS (median, not reached vs. 8.4 months; HR  .28; 95% CI,  .08–.84; *p* = .02) and OS (median, not reached vs. 19.2 months; HR  .18; 95% CI,  .04–.73; *p* < .01) than the non‐R0 group. In our study, the OS and PFS for the R0 group were significantly longer than non‐R0 group, which further confirmed that R0 resection serves as a critical prognostic factor. Our study demonstrated that immunochemotherapy did not improve the R0 resection rate compared to triplet chemotherapy in patients with BR‐ESCC. However, in this study, all patients underwent totally minimally invasive McKeown surgery, with no cases requiring conversion to thoracotomy or laparotomy, which exhibited superior surgical tolerability compared with triplet chemotherapy (92.3% of patients underwent the McKeown procedure).^7^ Compared to triplet chemotherapy, immunochemotherapy did not increased the postoperative complication rate (34.5% vs. 34.6%).^6^ In addition, immunochemotherapy demonstrates a higher pCR rate (18.0% vs. 8.5%), and postoperative pathological staging results revealed that a greater proportion of patients receiving immunochemotherapy were downstaged to stage I than those receiving triplet chemotherapy (69.0% vs. 26.9%).^6^ The observed enhancement in the pathological response may have the potential to translate into long‐term survival benefits for patients undergoing immunochemotherapy. However, additional follow‐up is necessary to substantiate this hypothesis. Future research on immunochemotherapy should prioritize the management of toxicity. Potential strategies include that using PD‐L1 inhibitors with more favourable safety profiles, adopting less toxic chemotherapy agents, or reducing the number of treatment cycles.

Several limitations of our trial should be considered when interpreting the results. First, our study was a single‐centre, single‐arm phase 2 trial with a small sample size. Second, our study did not include patients with oesophageal adenocarcinoma. Third, our study did not enrol patients over 76 years of age, and the optimal treatment for this population requires further investigation. In addition, the study repeatedly compared its data with previous studies; such comparisons lack rigorous statistical consideration and are for reference only. Consequently, the conclusions derived from this study should be interpreted with caution.

In conclusion, the regimen under investigation did not exhibit the anticipated statistical benefit in enhancing surgical conversion rates for BR‐ESCC. Nevertheless, the treatment strategy of induction immunochemotherapy followed by surgery resulted in significant tumour downstaging and a significant pathological complete response rate. Patients who achieved R0 resection exhibited improved survival outcomes. Subsequent studies should focus on optimizing the management of toxicity associated with induction therapy regimens.

## AUTHOR CONTRIBUTIONS

**Hong Yang**: study concept; study design; data interpretation. **Jia‐Di Wu**: data analysis; data interpretation; writing the paper. **De‐Shen Wang**: study concept; supervision. **Zhi‐Qiang Wang**: study concept; Supervision. **Qiao‐Qiao Li**: study concept; supervision. **Ren Chao**: writing review & editing. **Bin‐Yang Xu**: data curation. **Cai‐Yan Fang**: data analysis. **Zhi‐Chao Li**: data analysis. **Yan Huang**: data analysis. **Ji‐Yang Chen**: data curation. **Qiong Tan**: data curation. **Yu‐Hong Li**: study concept; study design; data interpretation.

## CONFLICT OF INTEREST STATEMENT

The authors declare no conflicts of interest.

## ETHICS STATEMENT AND CONSENT TO PARTICIPATE

The study received approval from the Ethics Committee of Sun Yat‐sen University Cancer Center. All patients included in the study provided written informed consent before enrolment.

## Supporting information



Supporting information
