# Supplementary material for: Sintilimab plus cisplatin and nab‐paclitaxel induction treatment for locally advanced borderline‐resectable oesophageal squamous cell carcinoma: A single‐arm, prospective, phase 2 study (NEOCRTEC2001)
Source: Clin Transl Med. 2026 May 11;16(5):e70691. doi: 10.1002/ctm2.70691 (PMC13159127; doi:10.1002/ctm2.70691)
Supplement: Supplementary file 1 — Supporting information [file CTM2-16-e70691-s001.docx]

**Inclusion and exclusion criteria**

Inclusion criteria include: 1) Patients are required to submit a signed Informed Consent Form. 2) Age ≥ 18 years old. 3) Patients must not have undergone any prior anti-tumor treatment for esophageal cancer, including surgery, chemotherapy, interventional therapy, immunotherapy, or radiotherapy. 4) Life expectancy > 3 months. 5) General physical status (ECOG PS SCORE): 0-1. 6) Blood routine test (within 7 days): Hb ≥9g/L, NE ≥1.5×10^9^/L, PLT ≥90×10^9^/L. 7) Liver and kidney function test (within 7 days): total bilirubin ≤1.5 UNL, creatinine ≤1.5 UNL, AST /ALT ≤2.5 UNL, ALP ≤5.0 UNL. 8) No serious complications such as active gastrointestinal bleeding, perforation, jaundice, intestinal obstruction, fever unrelated to malignant disease>38℃. 9) Patients with reproductive potential should take effective contraceptive measures. 10) Patients with good compliance and that can attend scheduled follow up to assess the efficacy and adverse reactions of the treatment.

Exclusion criteria include patients with cervical esophageal squamous cell carcinoma, distant metastasis, high risk of esophageal obstruction requiring intervention, or esophageal stent implantation. Other exclusions are concurrent primary cancers (except cured skin basal cell carcinoma and in situ cervical carcinoma), history of immunosuppressive drug use (excluding certain low-dose glucocorticoids), autoimmune diseases requiring treatment (unless controlled), primary immunodeficiency, active tuberculosis, organ transplantation history, interstitial lung disease requiring steroids, monoclonal antibody or chemotherapy allergies, severe heart disease, chronic diarrhea or renal insufficiency, active infections, cognitive or mental disorders, pregnancy or breastfeeding, and other conditions that may affect participant safety or study results.


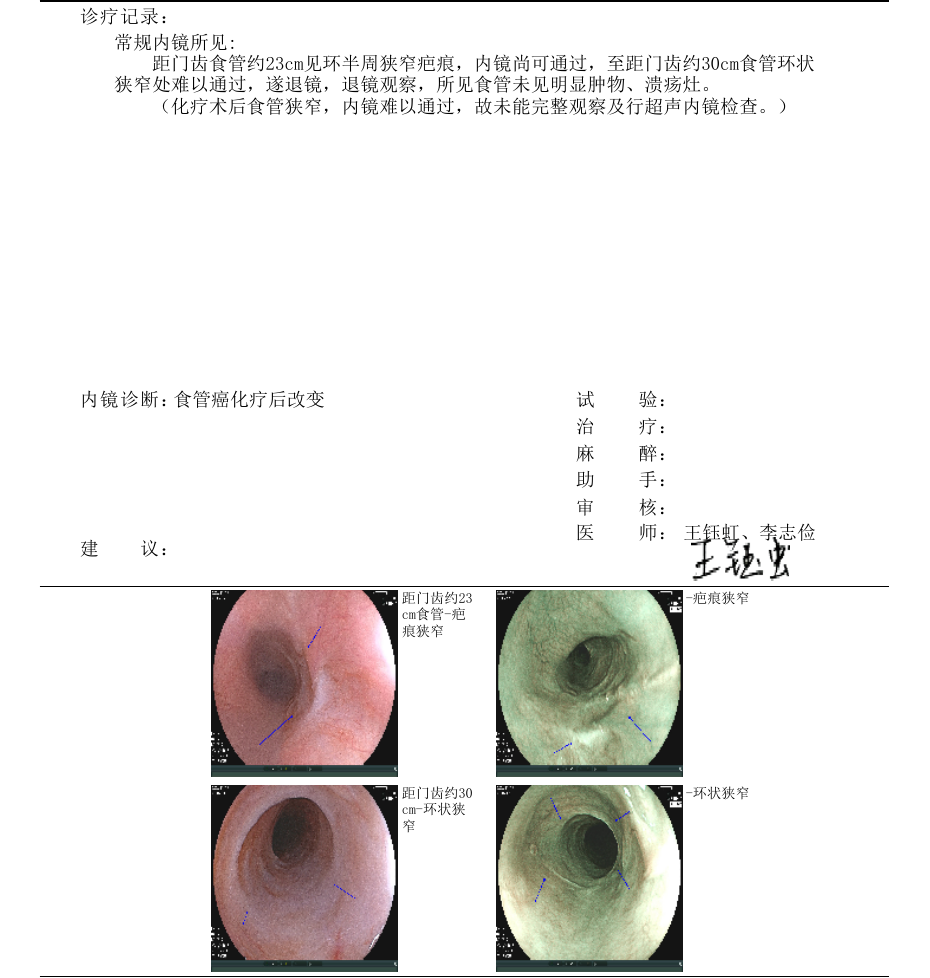

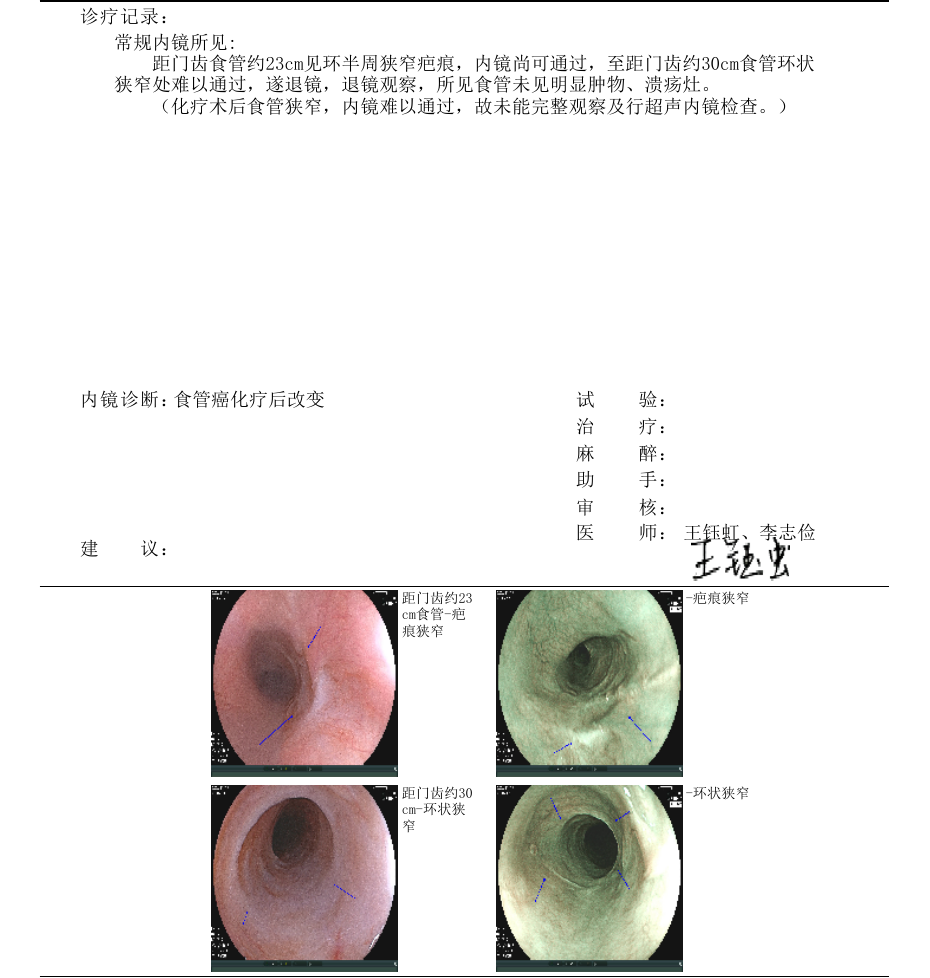

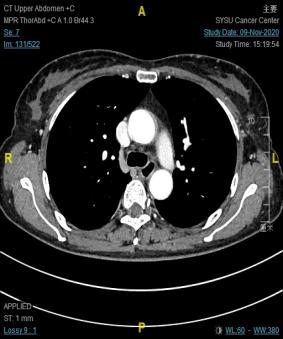

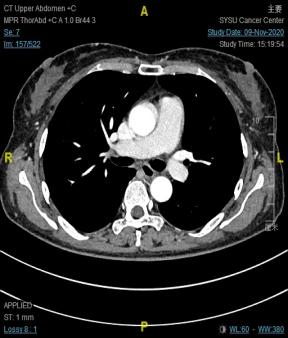

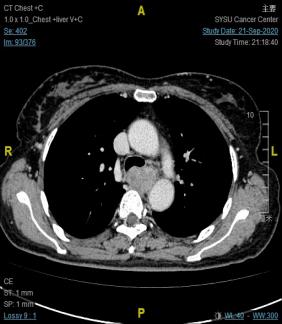

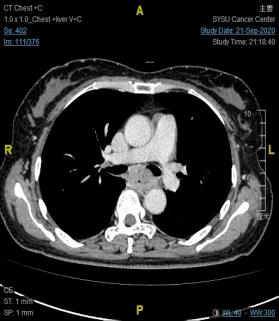

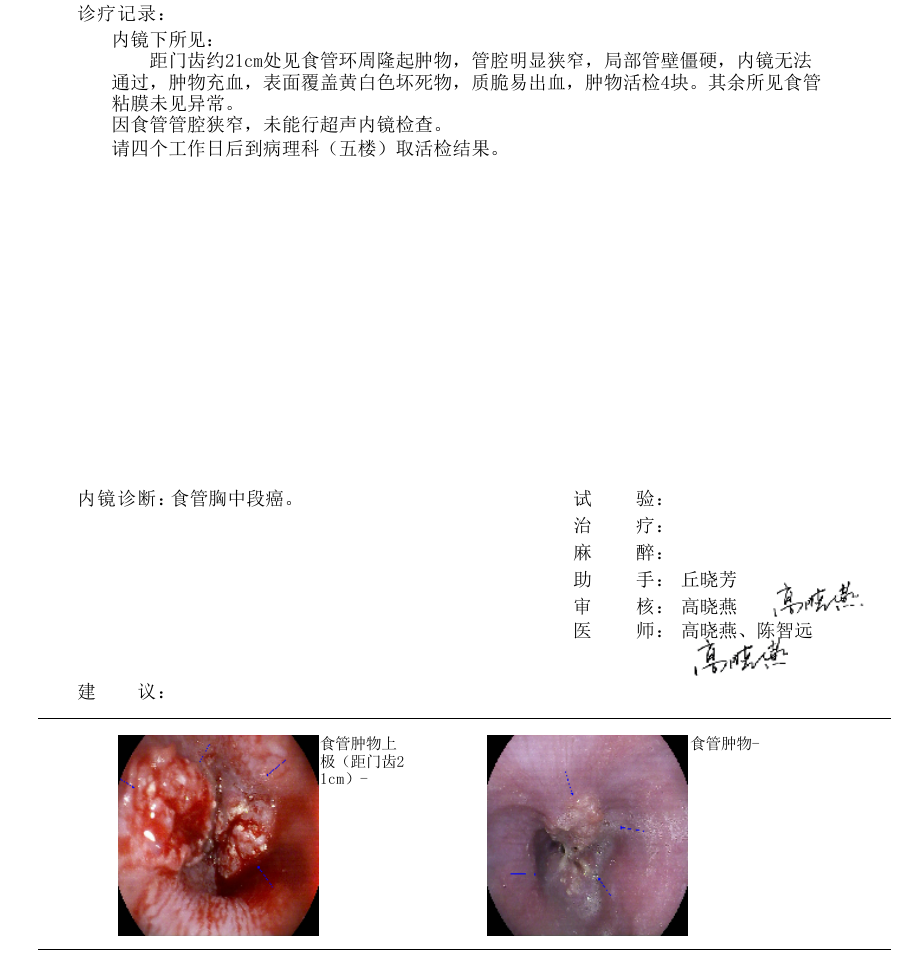

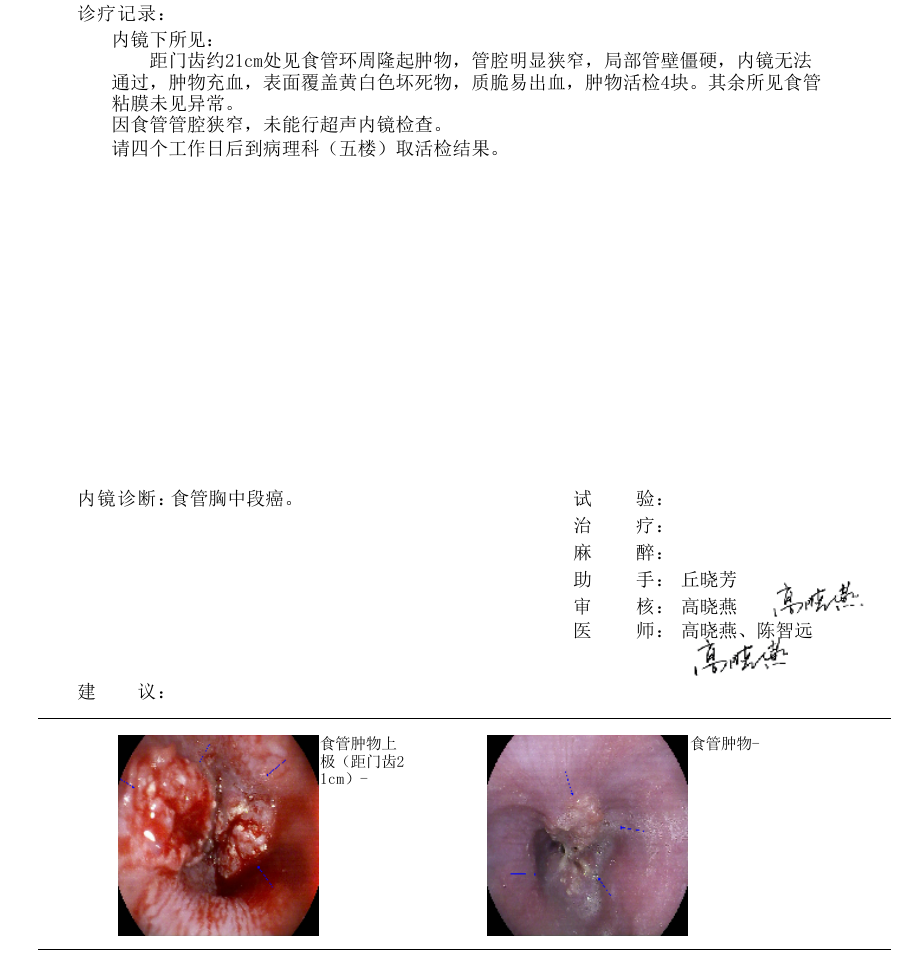


1. Before induction treatment B. After induction treatment

Supplementary Figure1. An example of BR-ESCC. The primary tumor was suspected to have invaded the airway, and causing severe esophageal stenosis. After induction immunochemotherapy, there was a marked reduction in tumor size, and the esophageal lumen was restored. Subsequent postoperative pathology confirmed a pCR.

| **Supplementary Table 1. postoperative complications** | | | | | |
| --- | --- | --- | --- | --- | --- |
| Postoperative complications | Total | Grade 1-2 | Grade 3 | Grade 4 | Grade 5 |
| Hemorrhage | 0 | 0 | 0 | 0 | 0 |
| Atelectasis | 2 (6.9%) | 2 (6.9%) | 0 | 0 | 0 |
| Arrhythmia | 4 (13.8%) | 4 (13.8%) | 0 | 0 | 0 |
| Heart failure | 1 (3.4%) | 1 (3.4%) | 0 | 0 | 0 |
| Chylothorax | 0 | 0 | 0 | 0 | 0 |
| Pleural effusion | 6 (20.7%) | 6 (20.7%) | 0 | 0 | 0 |
| Anastomotic fistula | 2 (6.9%) | 0 | 2 (6.9%) | 0 | 0 |
| Injury of recurrent nerve | 0 | 0 | 0 | 0 | 0 |
| Tracheal fistula | 1 (3.4%) | 0 | 1 (3.4%) | 0 | 0 |
| Pneumothorax | 4 (13.8%) | 4 (13.8%) | 0 | 0 | 0 |
| Pulmonary infection | 5 (17.2%) | 2 (6.9%) | 0 | 3 (10.3%) | 0 |
| Respiratory failure | 3 (10.3%) | 0 | 0 | 2 (6.90%) | 1 |
